# The International Temperance Congress

**Published:** 1901-04-20

**Authors:** 


### The Hospital

A Journal of

The Medical Sciences and Hospital Administration.

Vol. XXX.—No. 760.] EDITED BY SIR HENRY BURDETT, K.C.B., AND SOLOMON C. SMITH, M.D., M.R.C.P. [April 20, 1901.

### The International Temperance Congress.

To whatever extent it may be permissible to doubt whether a Congress be a machinery calculated to promote the establishment of truth, as contrasted with the predominance of strongly held opinion, that extent of uncertainty may, we think, be safely indulged in with reference to the International Temperance Congress which assembled last week at Vienna. Whatever opinions may be held about alcohol, there is practical unanimity, among all sensible men, with regard to the evils of intemperance. No Congress is required in order to obtain universal assent to the proposition that alcohol, like many things which are of unquestionable value to mankind—wealth, for example, or physical strength, or eminent intellectual capacity—is capable of being applied to unworthy and injurious purposes; or to the proposition that its misuse is attended by consequences far reaching in their diffusion and eminently disastrous in their character. But, just as neither the deceitfulness of riches, nor the brutalities of ruffianism, nor the perversions of great mental gifts have as yet succeeded in convincing mankind that the world would be improved by a universality of poverty, of weakness, or of stupidity, so there remain some who are unable to discover, in the wrongful use of alcohol, any evidence that it may not be used rightfully, or that its rightful use may not be attended by advantages commensurate with its acknowledged potency. Such people reflect, compare, analyse, judge. Frequently, even for long periods, the absence of conclusive evidence compels them to remain in a condition of suspended judgment, and to meet the vehemence of partisanship by Faraday's favourite formula, "It may be so." It was well said by Dugald Stewart that nearly all certainty of human knowledge is separated from the unknown by a zone, so to speak, of intermediate territory, a zone which is explored by the assistance of hypothesis and of experiment, and among the uncertainties of which men of science, in the proper sense of that much abused phrase, feel their way modestly and with circumspection. As between the people who denounce alcohol in all its forms, and those who believe that it may have its uses, even in the human economy, this belt of intermediate territory does not exist. The separation is only by a fence of barbed wire; and the forces ranged upon either side of it come to blows whenever they meet.

What is thus customary upon a small scale among ourselves, appears from the reports which have reached this country to have been repeated upon a larger one at Vienna. "The harmony manifested in the speeches delivered at the opening sitting" became, according to the *Times*, "somewhat less evident in the subsequent discussion;" an announcement pleasantly relieved by the addition that "the vigour of the debates had only increased public interest in the proceedings." A "frank apology" is said to have been made by a German physician, who accused his brethren of setting a bad example to their patients, and attributed "their" high death-rate (it does not appear whether that of doctors or patients) to the prevalence of drinking habits. We are further told that the possibility of "untoward consequences" had already been averted by a combination of humour and firmness on the part of the chairman, who, to judge from the former of these attributes, cannot himself be a teetotaller. It would seem, however, from subsequent portions of the report, that the firmness was even more required than the humour, for we read of "traces of electricity in the air," of "stern repression" by the chairman, and of his declaration that, although he should be sorry to resort to strong measures for the maintenance of order, "terrorism would not be tolerated." All this commotion is said to have arisen over purely technical and scientific questions in relation to the pathological and other effects of alcohol; and the excitement appears to have faded away when expedients for the diminution of drunkenness came under consideration. The most practical of such expedients were those in operation in the French army as described by Dr. Richard, the physician to the military hospital in Paris, and they originated in ordinances issued by the Ministers of War, Generals de Gallifet and André. They consist chiefly in restrictions upon the sale of drinks in the canteens, and in the delivery of lectures on the dangers of drink by officers or military surgeons. Papers were also read by Count Skarzynski, of Warsaw, describing the effects produced by the introduction of a State monopoly of distillation in Russia, and by the establishment of popular entertainments on Sundays and holidays, organised by the Government for the purpose of diminishing the temptations incidental to such occasions. The Count's papers seem however to have been mercilessly pulled to pieces by a Polish lady doctor, whose "somewhat acidulated remarks" were received with much applause by the audience. It is manifest that Russian official statistics do not command entire assent at Vienna.

### The Marriage of the Unfit.

While we quite agree with all that can be said against the laxity which now prevails in too many civilised countries in regard to the marriage of the unfit, we cannot feel so much confidence as some do in the remedies proposed. That epileptics should be allowed to marry and produce their kind, that imbeciles should perpetuate their species, and that diseased persons should enter into holy matrimony is all very wrong. At the same time it is difficult to see how the evil consequences of these unions are to be prevented, and certainly the attempts to control them by such laws as have lately found favour in certain States of America are hardly likely to have the desired effect. In several States laws have been passed with the object of preventing the marriage of persons suffering from certain forms of diseases and certain states of mental disorder, and if there were any hope of their being effective in preventing not only the marriage but the union of such persons laws to this effect would have our every sympathy. When, however, it becomes a question of demanding medical certificates testifying to the health of the applicants and their fitness for marriage, we rather shrink from the responsibility thus sought to be imposed upon the medical profession. We may be permitted indeed to doubt whether any physician, or any such board of physicians as is contemplated in Minnesota, for example, would find it practicable to establish any acceptable standard of fitness for marriage which would on the one hand be effectual, and on the other would be generally accepted. Take the two cases which more than any excite the indignation of thinking people, that of the man who infects his wife or his offspring with diseases resulting from his own licentiousness, and of the man or woman afflicted with epilepsy or mental disorder who passes on to succeeding generations the neuropathic taint. It is easy to say that such a thing should be prevented by law. But when one tries to lay down a rule on the subject, what endless difficulties stand in the way! No one who has watched the various changes of opinion which have followed the growth of knowledge concerning these diseases can pretend that there is any criterion by which admission within the pale of matrimony could be safely regulated without excluding so many as to set up a large and influential caste of compulsory celibates—a dangerous institution. As to epilepsy and chronic insanity, again, the matter is by no means as simple as it looks. It would be a safe and a good rule no doubt for those so afflicted to be debarred from perpetuating their species. But that would not be enough. The family taint exists in others besides the one who shows the symptom, and if the result aimed at is to be attained they also must be excluded from the pale of matrimony! According to the Bill now proposed in Minnesota a physician must certify not only to the saneness of mind of the contracting parties, but to the soundness of intellect of both their families, and the minister who marries people without such a certificate is liable to fine, or even to imprisonment. The proposals are impracticable, good as may be their intent. To render illegal the marriage of the obviously insane or the actively diseased is good, but perhaps hardly worth while in view of the infrequency with which such marriages take place. To attempt, on the other hand, such a widespread interference with marriage as should exclude all those who bore the taint would not only be a big affair, but would in practice so largely depend upon medical certificates regarding matters as to which medical knowledge is by no means positive that its chief direct effect would be to give occupation to the lawyers. Indirectly, moreover, such a scheme would be certainly productive of many evils. No State can debar from marriage a large section of its people without running the risk of marriage itself becoming a matter of indifference.

### Free Baths.

We have many public baths in England where for a very small sum a person may get a good scrub. It is probable that in some cases the bath costs more than is paid for it, but it by no means follows from this that the obligation is entirely on the side of the workman; in fact, it might be fairly argued that every dirty person does his town a benefit whenever he renders himself clean. Perhaps this is the view that has influenced the people of Buffalo in instituting free baths. At any rate the municipality of that city runs a free public bath, open all the year round for both adults and children. Each bather is provided with a piece of soap and a clean towel free of charge, and so popular has the system become that a second establishment has been constructed in which shower baths also are extensively provided. That nearly eleven thousand persons should have availed themselves of these free baths during the month of January shows what an amount of work they are doing—a sanitary benefit which is by no means confined to the bathers, and one which is probably well worth what it has cost the municipality. A very large amount of money has been spent in England in the erection of public swimming baths, all of which is good, for swimming is a healthy exercise. But whether the cost to the public entailed by the support of these institutions would not be better spent in giving free scrubs to the unwashed is another question. Swimming is to a large extent a mere amusement, indulged in by quite a small proportion of the population, and the public advantage of which is comparatively small. Washing the unwashed, on the other hand, is directly or indirectly a benefit to every ratepayer, and one for which he may well expend the small sum necessary to ensure its being well done.

### Dr. Collingridge on Rats.

The most interesting part of the recently issued half-yearly report of the medical officer to the Port of London is that headed "Plague and Rats," in which, after a careful review of what is known as to the causal relation between rats and the spread of the plague, an account is given of the measures which have been taken at the various docks to diminish the danger from this cause, and suggestions are made as to future action in this direction. Dr. Collingridge quotes from the report of the German Plague Commission to show that not only does feeding with the smallest quantity of a plague bacillus cultivation invariably produce in the rat a fatal attack of the disease, but that these animals, when in a state of freedom, are accustomed to gnaw the bodies of their companions dead of plague, so that the pestilence spreads very quickly among them. Probably in any place that becomes affected some of the rats are killed by the disease and the rest migrate, and it is precisely in this migration of the rats that the danger exists. It cannot be said that the answers returned by the various dock companies as to their precautions regarding rats are altogether satisfactory. A few rat-catchers are employed, poison is put down here and there, and a considerable number of cats—amounting at one of the dock premises to two hundred—are kept with the object of keeping the rats down; but it seems that no serious attempt is made to exterminate them. As is stated by the officials at one dock the rat-catcher "receives the complaints of warehouse keepers" and "gives first attention to the places specially complained about." Evidently it is only when rats become a trouble that they are interfered with. Some of the docks appear to be absolutely swarming with rats. At Millwall 6,470 rats were destroyed last year, but it is admitted that in proportion to the numbers present the number caught was "very small." At the Royal Victoria Dock and the Royal Albert Dock the Company employ rat-catchers who destroy the rats by poisoning "In the case of any particular place being found to be infested with them, that place receives extra attention till the rats disappear," which would seem to show that by dint of such "extra attention" all round a general clearance might quite well be made. We cannot but feel that this matter is one of considerable gravity, and that means ought to be taken to enforce a much more effective war against rats than has hitherto been undertaken. The companies should be compelled to keep their premises clear of animals whose presence is not only a nuisance to all concerned but a danger to the community.

### Work for Lady Sanitary Inspectors.

Now that ladies are so frequently appointed to the post of sanitary inspector, health departments perhaps feel that they may properly endeavour to regulate various details of domestic management with which they would hardly have cared to interfere so long as they were dependent on "mere men" to see to the carrying out of their behests. At any rate, so only can we explain the temerity of the Health Committee of the Sunderland Corporation who, subject, of course, to confirmation, have adopted some new bye-laws which attempt to regulate various matters of domestic management. One of the clauses is as follows: "Every occupier in a tenemented house shall cause the floor of every room which has been let to him to be thoroughly swept once a day before the hour of 12 o'clock noon, and to be thoroughly washed once at least in every week, namely, Friday or Saturday, before 12 o'clock noon, and from time to time as often as may be necessary for the purpose of keeping the same in a clean and wholesome condition." Another clause provides that "Every window of every sleeping-room must be opened freely for, at least, an hour in the morning and an hour in the afternoon—unless the weather is bad or cases of sickness render this unwise." These are most admirable regulations and if enforced will doubtless add greatly to the healthiness of the poorer quarters of the town. Indeed, they might well be made to include houses far above tenements. But to enforce them! We do not envy the inspectors, and we sincerely hope that they are women. *Place aux dames!* Were we sanitary inspectors we should willingly yield precedence.

### The Colour Problem.

There are many indications that when once the settlement of South Africa comes practically before us as a question of the moment the race problem will assume a far more important position than it has so far been allowed to occupy. Nothing can be more extraordinary than the mixture of races which is at present to be met with in the streets of Capetown, and when it becomes necessary for us seriously to decide what is to be the status of these various non-European peoples, ranging as they do through every shade of colour and including emigrants from almost every land, we shall have a problem which will be hard to solve, and all the more hard from the fact that in its solution we in England, with no personal experience or knowledge of the colour difficulty, will have to take a part and bear much of the responsibility. One has to live in a country where the question of colour is constantly acute to understand how it enters into every problem of both private and public life, and it is much to be feared that very acute differences will arise between the English view, as voiced by Exeter Hall, and the Colonial view as voiced by those who push the negro off the footpath and deny him access to the tramcar. Nothing could more forcibly show the importance and the apparent insolubility of this problem of colour than the movement which is beginning to take voice in America in favour of the expulsion of the negro from the Southern States. The race difficulty there is always with them, and in some parts of the South it is a constant nightmare. American visitors to England, free for a time from the incubus, are not very willing to talk about it. It is, as they know, a blot on that liberty and equality which they love to regard as the very basis of their constitution. But the frequent lynchings which take place where blacks and whites live together in apparent amity, and the well-known state of social degradation and isolation in which the blacks are compelled to live, even where they are so numerous as to have obtained large political power, shows how impassable, and as we venture to think, properly impassable, is the gulf which divides the black man from the white, and how merely superficial is the pretence that all men are equal within the borders of the Union. In America it has long been felt to be a sanitary as well as a social question, and in South Africa it is the sanitary view, as emphasised by the measures taken for the control of the plague, that has lately brought the matter more prominently before our notice. No nation can afford to maintain in its midst an inferior race, a mass of people living under inferior conditions and not conforming to those sanitary requirements which are found essential if a high standard of public health is to be maintained. Yet that is what has happened wherever the negro and the white man have attempted to live side by side; and when in a practical and progressive country like America, with all its experience of the various aspects of the colour problem, we find that a demand is arising for the expulsion of the negro—truly a policy of despair—as the only possible and permanent solution of the difficulty, we cannot but feel that the task of ensuring social and sanitary safety without interfering with those broad principles of equality which look so well in theory and yet work out so curiously in practice, will be by no means the least of the many troubles which we have to face in South Africa.

